# Ten-year risk of second primary malignancies among chemotherapy-treated early-stage breast cancer survivors: a multicentre cohort study from the Turkish Oncology Group

**DOI:** 10.3389/fonc.2026.1756315

**Published:** 2026-07-07

**Authors:** Haydar Cagatay Yuksel, Elvina Almuradova, Ozge Buyukahiska, Bahiddin Yilmaz, Caner Acar, Gokhan Sahin, Salih Tunbekici, Sercan On, Hikmet Akar, Mustafa Sahbazlar, Ertugrul Bayram, Muzeyyen Asli Ergozoglu, Besire Nurdan Tazebay, Devrim Cabuk, Sait Kitapli, Bedriye Acikgoz Yildiz, Sedat Yildiz, Ramazan Cosar, Tugba Akin Telli, Mesut Yilmaz, Ilhan Hacibekiroglu, Teoman Sakalar, Nargiz Majidova, Gordana Petrovska, Gulcin Celebi, Mehmet Emin Kalender, Meltem Rahman, Cengiz Akosman, Yudagul Danaci, Erdinc Nayir, Pinar Gursoy, Erhan Gokmen

**Affiliations:** 1 Division of Medical Oncology, Ege University Faculty of Medicine, Izmir, Türkiye; 2 Division of Medical Oncology, Can Hospital, Izmir, Türkiye; 3 Division of Medical Oncology, Ondokuz Mayıs University Faculty of Medicine, Samsun, Türkiye; 4 Division of Medical Oncology, Manisa Celal Bayar University Faculty of Medicine, Manisa, Türkiye; 5 Division of Medical Oncology, Cukurova University Faculty of Medicine, Adana, Türkiye; 6 Division of Medical Oncology, Kocaeli University Faculty of Medicine, Kocaeli, Türkiye; 7 Division of Medical Oncology, Mugla Sitki Kocman University Faculty of Medicine, Mugla, Türkiye; 8 Division of Medical Oncology, Pamukkale University Faculty of Medicine, Denizli, Türkiye; 9 Division of Medical Oncology, Afyonkarahisar Health Sciences University, Afyonkarahisar, Türkiye; 10 Division of Medical Oncology, Demiroglu Bilim University and Group Florence Nightingale Hospital, Istanbul, Türkiye; 11 Division of Medical Oncology, Faculty of Medicine, Sakarya University, Sakarya, Türkiye; 12 Division of Medical Oncology, Kahramanmaraş Necip Fazıl City Hospital, Kahramanmaraş, Türkiye; 13 Division of Medical Oncology, Marmara University Faculty of Medicine, Istanbul, Türkiye; 14 Division of Medical Oncology, University Clinic for Radiotherapy and Oncology, Skopje, North Macedonia; 15 Department of Internal Medicine, Ege University Faculty of Medicine, Izmir, Türkiye; 16 Department of Medical Oncology, Meddem Hospital, Isparta, Türkiye; 17 University of Health Sciences, Tepecik Education and Research Hospital, Izmir, Türkiye; 18 Division of Medical Oncology, Medicalpark Hospital, Ordu, Türkiye; 19 Division of Medical Oncology, İnönü University Faculty of Medicine, Malatya, Türkiye; 20 Department of Medical Oncology, Mersin Medical Park Hospital, Mersin, Türkiye

**Keywords:** breast cancer, incidence, multiple primary cancer, risk, second primary cancer

## Abstract

**Purpose:**

As survivorship improves, second primary malignancies (SPMs) have become a clinically relevant late effect of localised breast cancer treatment. Evidence from the Middle East and Balkans is scarce, and contemporary data from Türkiye are lacking.

**Materials and methods:**

We conducted a rigorous retrospective, multicentre cohort study within the Turkish Oncology Group across 14 tertiary centres. The medical records of 6, 552 women with early-stage breast cancer (2008–2022) were meticulously reviewed. SPMs were defined according to IARC/SEER multiple-primary rules, and only the first SPM per patient was included. We calculated standardised incidence ratios (SIRs) with exact Poisson 95% confidence intervals (CIs) using age- and period-specific national female incidence rates averaged over the 2010–2020 period. Person-years were stratified by attained age and calendar period to ensure the accuracy and reliability of our results.

**Results:**

During a median follow-up of 5.4 years (interquartile range, 1.1–9.4), 174 women developed pathologically confirmed SPMs. Breast cancer survivors had a significantly higher SPM risk compared to the general population (SIR, 1.66; 95% CI, 1.42–1.93). Excluding breast primaries, the excess remained (SIR 1.78; 95% CI, 1.51–2.09). The most significant increases were observed for thyroid cancer (SIR 7.09; 95% CI, 4.45–10.70) and sarcomas (SIR 7.14; 95% CI, 3.43–13.10), followed by ovarian (SIR 4.35; 95% CI, 2.58–6.89) and pancreatic cancers (SIR 4.08; 95% CI, 1.11–10.50). Risks for colorectal, brain, bladder, kidney, gastric, and contralateral breast cancers did not differ from expectations (all p>0.05).

**Conclusion:**

Turkish breast cancer survivors face a markedly increased SPM risk, especially for thyroid, sarcoma, and ovarian cancers. These findings should be interpreted cautiously in the context of treatment selection, surveillance intensity, hereditary predisposition, and regional background cancer risks. Prospective population-based studies incorporating detailed treatment exposures, germline testing, and standardized follow-up data are needed to clarify the mechanisms underlying these associations.

## 1 Introduction

Breast cancer is the most commonly diagnosed cancer among women worldwide and remains a significant cause of cancer-related death (1). Recent improvements in early detection and multimodal treatments (surgery, systemic therapy, radiotherapy) have significantly improved breast cancer survival rates (2). Consequently, the number of breast cancer survivors continues to increase (3). As of 2022, there were approximately 4 million breast cancer survivors in the United States, and this figure is projected to increase by about 50% by 2040 (4).

As survival rates for breast cancer continue to improve, clinical focus has shifted from acute treatment toward long-term survivorship, with particular attention to the late effects of therapy. Among these, second primary malignancies (SPMs) represent one of the most significant and clinically relevant complications (5). Such malignancies may result from treatment-induced toxicities, inherited genetic predispositions, or environmental and lifestyle influences, representing a major contributor to long-term morbidity and mortality in cancer survivors (5–7). Several epidemiological studies have shown that breast cancer survivors have a higher risk of developing a SPM compared to the general population, with higher incidences observed in thyroid, ovarian, uterine, haematological cancers, and sarcomas (8–11).

Most existing data on SPMs following breast cancer are derived from Western and East Asian populations, with a notable paucity of evidence from the Middle East and neighbouring regions, including Türkiye. To date, apart from a survival analysis by Gulhan et al. in 2009, which focused on gynaecologic cancers, no comprehensive data have been reported (12). Elucidating the incidence patterns and clinicopathologic features of SPMs is crucial for developing tailored survivorship care plans and implementing evidence-based surveillance protocols.

In this context, we conducted a retrospective, multicenter cohort study within the Turkish Oncology Group to assess the incidence and clinicopathologic characteristics of SPMs among chemotherapy-treated early-stage breast cancer survivors who had completed primary treatment. The primary aim of this study was to compare the risk of SPMs in this population with that of the general population and to describe their clinicopathologic features. To our knowledge, this is the first comprehensive study to quantify SPM risk among breast cancer survivors in Türkiye. By filling this critical gap, our study provides large-scale, real-world data that can support the development of tailored survivorship care plans and guide evidence-based strategies for long-term follow-up of breast cancer survivors.

## 2 Materials and methods

This retrospective, multicentre cohort study, conducted by the Turkish Oncology Group across fourteen tertiary oncology centres, included female patients who had completed primary treatment for early-stage breast cancer between 2008 and 2022. Medical records of 6, 552 women were screened using institutional cancer registries and electronic medical records. SPMs were defined as histologically distinct neoplasms, confirmed by pathology reports, and coded according to the International Classification of Diseases for Oncology (ICD-O).

**Figure 1:**
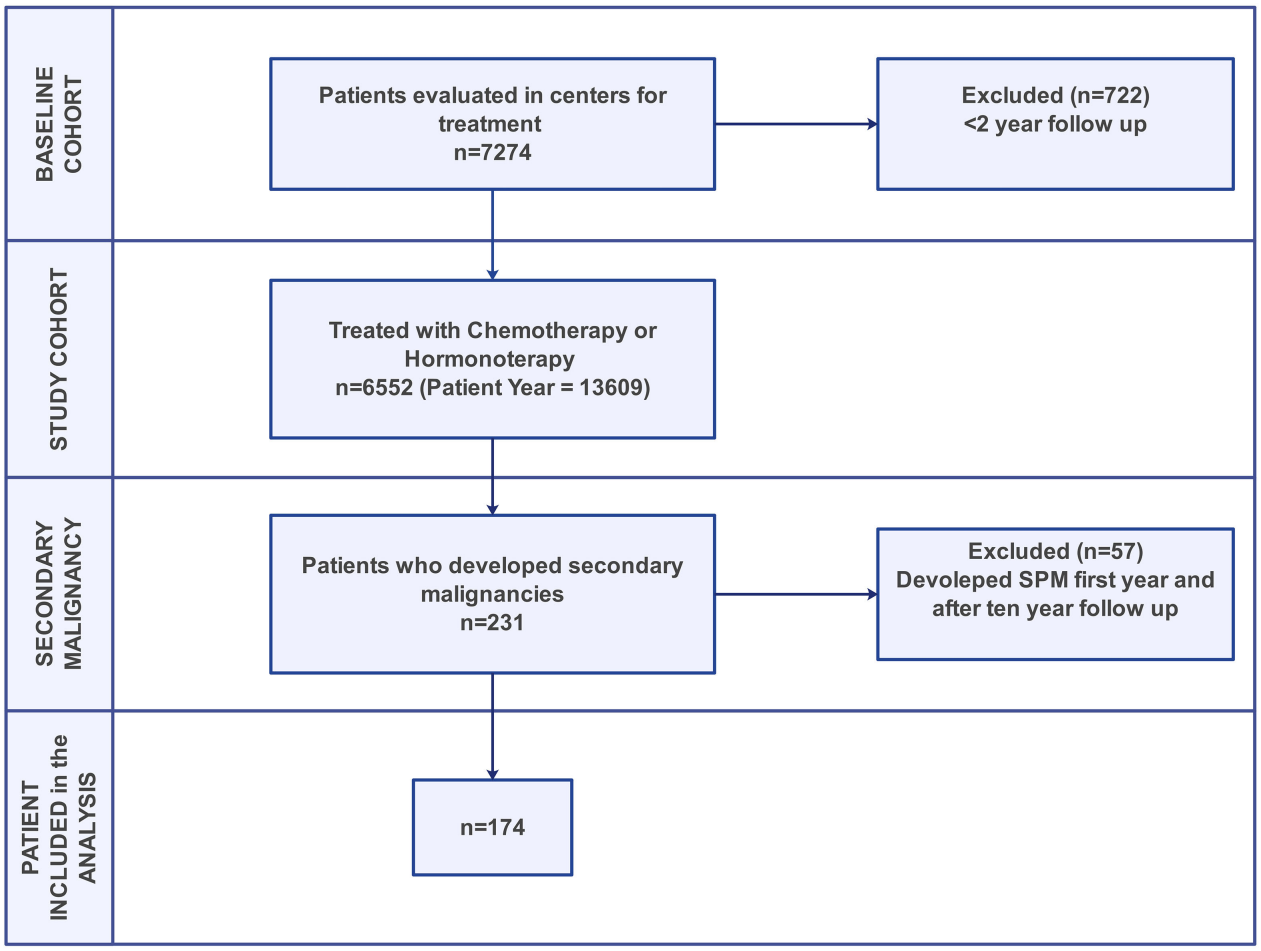
Description of the study population SPM: Secondary primary malignancy. Patient-years were calculated from the date of initial treatment to the last follow-up or the occurrence of SPM. Patients with >1 years of follow-up or with SPM diagnosed within the first year and after ten years were excluded from the analysis.

A total of 231 patients were initially identified as having developed a SPM during routine follow-up visits, which typically included clinical examination every 3–6 months for the first five years and annually thereafter, along with imaging as clinically indicated. SPMs were defined according to the IARC/SEER multiple-primary rules. According to these criteria, tumours were considered distinct primary malignancies if they arose in different anatomical sites or, when occurring in the same organ, exhibited different histological characteristics. Metastases, recurrences, and progression of the primary tumour were not classified as SPMs. For the primary analysis, we counted only the first incident SPM per woman. We excluded male patients and women diagnosed before age 30 or at 80 years or older to focus on the typical adult female breast cancer survivor population. We also excluded patients with metastatic disease at diagnosis, those who did not have surgery within one year of diagnosis, and cases where the SPM was detected less than one year post-surgery (to eliminate synchronous tumours) or beyond ten years of follow-up. During routine follow-up, breast cancer survivors were generally monitored with clinical assessment and annual mammography in accordance with contemporary guideline recommendations (13). In addition, chest radiography, abdominal ultrasonography, and serum tumor marker measurements were frequently used in routine clinical practice across participating centers, reflecting real-world surveillance patterns in Türkiye. We chose a ten-year follow-up limit because, in many cases, patients are discharged from oncologic surveillance after a decade, and long-term follow-up tends to be inconsistent in middle-income settings such as Türkiye. Restricting the analysis to this period, therefore, allowed a more reliable capture of SPMs during routine follow-up (**Figure 1**). This design specifically aimed to evaluate SPMs occurring within the clinically relevant surveillance window of breast cancer survivorship. Detailed treatment-related variables, including radiotherapy dose, treatment fields, and exposure to specific chemotherapeutic agents (e.g., alkylating agents), were not available in the dataset, limiting treatment-specific risk analyses. Due to limitations in the availability of genetic data, BRCA mutation status could not be retrieved or systematically evaluated for the study population.

To compare cancer risk among breast cancer survivors with that of the general population, we utilized data from the Turkish Annual Cancer Statistics Reports published by the Ministry of Health. Age-specific cancer incidence rates for the general female population were obtained from reports covering the years 2010–2020. To improve the stability of the estimates and minimize the impact of year-to-year variability, incidence rates were calculated using the average values across these consecutive years. This approach was chosen to ensure more stable and representative population-level incidence estimates over time while aligning with the cohort’s study period. All data were derived from publicly available national registry sources (14). Given that our cohort included recent cases, we calculated the average incidence rates for the 2010–2020 period to estimate standardised incidence ratios (SIRs) along with their corresponding 95% confidence intervals (CIs). Person‐years at risk were calculated from the date of primary breast cancer diagnosis until death, diagnosis of a SPM, or last hospital contact. The median follow-up time from breast cancer diagnosis to either SPM diagnosis or last contact was 5.4 years (IQR, 1.1–9.4), with a total of 13, 609 person-years at risk accrued by the cohort. Expected numbers of cancer cases were calculated by multiplying stratum-specific person-years at risk (defined by attained age and calendar period using the Lexis approach) by the corresponding age-specific cancer incidence rates in the general female population. Standardized incidence ratios (SIRs) were computed as the ratio of observed to expected cases (O/E), with 95% confidence intervals estimated assuming a Poisson distribution. An SIR was considered statistically significant when its 95% confidence interval did not include 1.00.

To further examine the potential contribution of surveillance bias, national reference data for median age at diagnosis of the major SPMs (breast (15), lung (16), colon (17), ovary (18), endometrium (19), thyroid (20)) were extracted from published multicentre and nationwide studies, whereas reference distributions for localized, regional, and distant stage at diagnosis were obtained from the Turkish National Cancer Statistics (14). Age comparisons were descriptive, and three-category stage distributions in the survivor cohort were compared with national reference distributions using one-sample goodness-of-fit tests.

Because age-stratified Turkish population-level stage distributions were unavailable, an additional exploratory age-adjusted stage analysis was performed using age-specific stage distributions obtained from the SEER database (41). Patients were stratified according to age at SPM diagnosis (<50, 50–64, and ≥65 years). Expected numbers of localized, regional, and distant-stage cancers were calculated by applying age-specific reference proportions to the observed number of SPMs within each age stratum. Observed and expected stage distributions were compared descriptively using observed-to-expected ratios. Given the absence of individual-level comparator data and the use of an external reference population, these analyses were considered exploratory and were primarily undertaken to assess the potential impact of surveillance bias.

Comparisons of clinicopathological characteristics among cancer subtypes were performed using the Chi-square or Fisher’s exact test for categorical variables and one-way ANOVA (Welch’s correction) or the Kruskal–Wallis test for continuous variables, as appropriate. Normality of continuous variables was assessed using the Shapiro–Wilk test. A two-sided p-value <0.05 was considered statistically significant. Cases with missing data were excluded from the respective analysis. All statistical analyses were performed using R software (version 4.4.2; R Foundation for Statistical Computing, Vienna, Austria).

Ethical approval for the study was obtained from the Ege University Ethics Committee (2024-2947 24-5.1T/39), and the study was conducted in accordance with the principles outlined in the Declaration of Helsinki.

## 3 Results

The baseline clinicopathological characteristics of the 174 patients included in the study are summarised in **Table 1**. The median age at breast cancer diagnosis was 62 years (interquartile range, 53.0–68.0). Most patients presented with T2 stage tumours (63.2%) and N1 nodal involvement (36.8%), with the majority classified as stage II disease. Invasive ductal carcinoma (IDC) was the predominant histological subtype, observed in 87.9% of cases. HER2 overexpression was detected in 23.0% of patients, while 79.3% were hormone receptor–positive. Chemotherapy was administered to all of the patients, most commonly with an anthracycline and taxane-based regimen. Additionally, 80.0% of patients received hormonal therapy, and 73.0% underwent radiotherapy.

**Table 1:** Baseline clinicopathological characteristics of the study cohort (N = 174).

Characteristic	n (%) or median (IQR)	Characteristic	n (%)
Age		HER2 status	
Median (IQR)	62.0 (53.0-68.0)	HER2 positive	40 (23.0)
T stage		HER2-Low	20 (11.5)
T1	59 (33.9)	Negative	114 (65.5)
T2	110 (63.2)	ER status (n=165)	
T3	5 (2.9)	Negative	27 (19.5)
N stage		Positive	140 (80.4)
N3	13 (7.5)	Chemotherapy regimen	
N2	15 (8.6)	AC - Taxan	75 (43.1)
N1	64 (36.8)	AC	55 (31.6)
N0	82 (47.1)	FEC	22 (12.6)
Stage		Docetaxel Carboplatin	18 (10.3)
Stage I	33 (19.0)	Taxan	5 (2.8)
Stage II	111 (63.8)	Endocrine therapy	
Stage III	30 (17.2)	AI/TMX	140 (80.4)
Grade		No	34 (19.5)
Grade 1	8 (4.6)	Anti-HER2 (n=40)	
Grade 2	98 (56.3)	Trastuzumab	29 (16.7)
Grade 3	68 (39.1)	Trastuzumab/ Pertuzumab	11 (6.3)
Histological type		RT (n=168)	
IDC	153 (87.9)	Yes	127 (73.0)
ILC	11 (6.3)	No	41 (23.6)
Medullary	2 (1.1)		
Mucinous	5 (2.9)		
Other	3 (1.7)		

Data are presented as n (%) unless otherwise indicated. Percentages may not total 100% due to rounding. Hormone receptor (ER) and HER2 status were assessed according to the ASCO/CAP guidelines, and tumour staging was performed based on the AJCC 7th edition staging system. IQR, interquartile range; IDC, invasive ductal carcinoma; ILC, invasive lobular carcinoma; HER2, human epidermal growth factor receptor 2; ER, estrogen receptor; PR, progesterone receptor; AC, adriamycin–cyclophosphamide; FEC, fluorouracil–epirubicin–cyclophosphamide; AI, aromatase inhibitor; TMX, tamoxifen; RT, radiotherapy.

**Figure 2:**
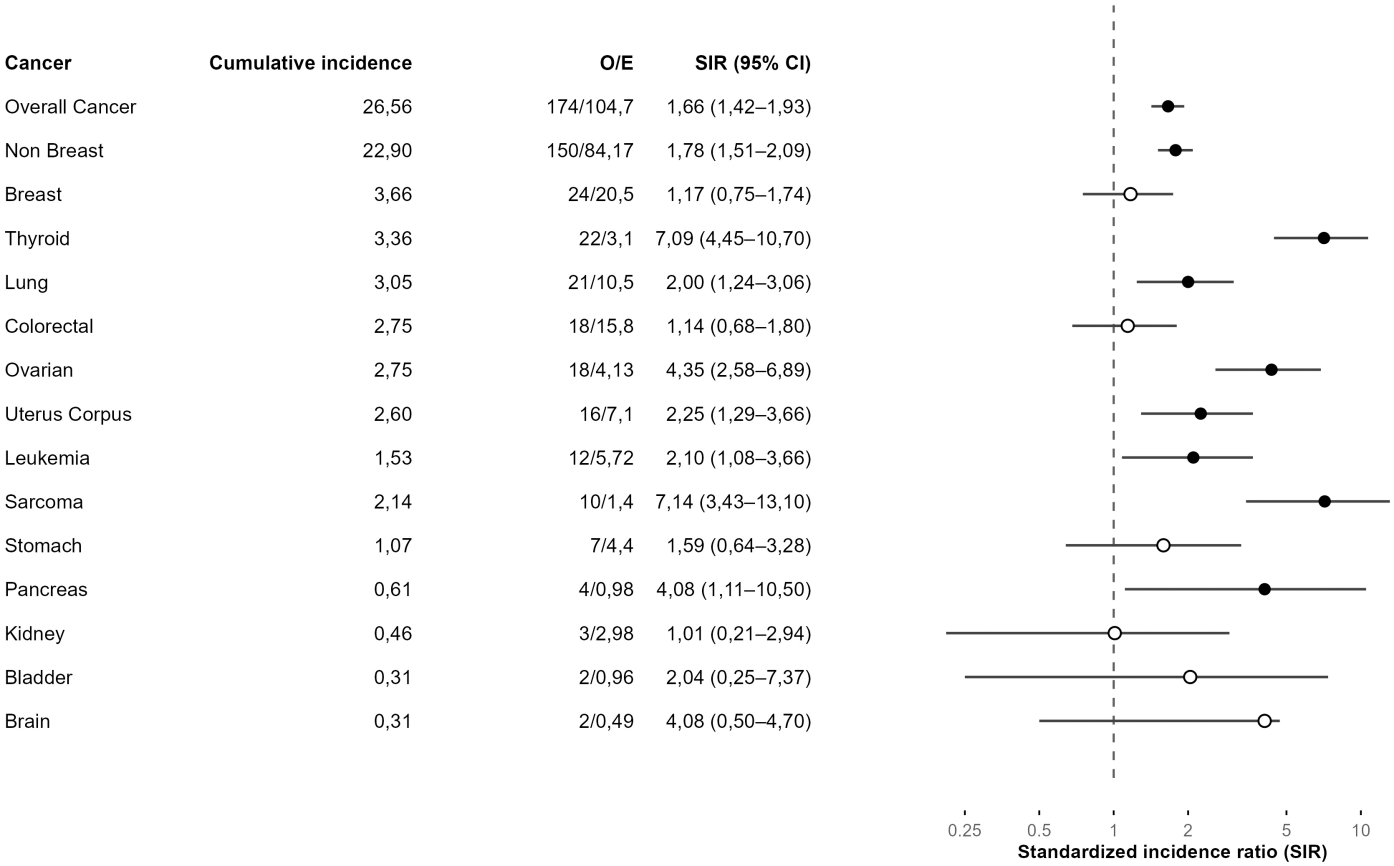
Risk of second primary cancer among women in the Turkish oncology group cohort. Abbreviations: CI, confidence interval; SIR, standardised incidence ratio. Observed and expected numbers are derived from registry data; SIR values and 95% CIs were calculated using exact Poisson tests. The dashed vertical line indicates the reference value of SIR = 1.0. Filled circles represent statistically significant associations (95% CI not crossing 1.0), whereas open circles indicate non-significant estimates. Cumulative incidence values are presented per 1, 000 patients.

Site-specific SIRs were estimated for 15 cancer sites (**Figure 2**). During the follow-up period, SPMswere identified across 15 distinct cancer sites, with a total of 174 (% 2.6%) cases observed. This corresponded to a combined standardised incidence ratio (SIR) of 1.66 (95% CI, 1.42–1.93) compared with the general population. Excluding breast cancer, the incidence of non-breast second primaries was 78% higher than expected (SIR, 1.78; 95% CI, 1.51–2.09). The highest excess risk was observed for thyroid cancer (SIR 7.09; 95% CI, 4.45–10.70) and sarcoma, including both soft tissue and bone (SIR 7.14; 95% CI, 3.43–13.10). Elevated risks were also seen for ovarian cancer (SIR 4.35; 95% CI, 2.58–6.89) and pancreatic cancer (SIR 4.08; 95% CI, 1.11–10.50). Moderate increases were noted for uterine corpus cancer (SIR 2.25; 95% CI, 1.29–3.66), leukaemia (SIR 2.10; 95% CI, 1.08–3.66), and lung cancer (SIR 2.00; 95% CI, 1.24–3.06). In contrast, no statistically significant excess risk was detected for brain (SIR 4.08; 95% CI, 0.50–4.70), bladder (SIR 2.04; 95% CI, 0.25–7.37), kidney (SIR 1.01; 95% CI, 0.21–2.94), colorectal (SIR 1.14; 95% CI, 0.68–1.80), contralateral breast (SIR 1.17; 95% CI, 0.75–1.74), or gastric cancers (SIR 1.59; 95% CI, 0.64–3.28) These findings are summarized and illustrated in the forest plot (**Figure 2**), which displays the SIR estimates with corresponding 95% confidence intervals.

**Figure 3:**
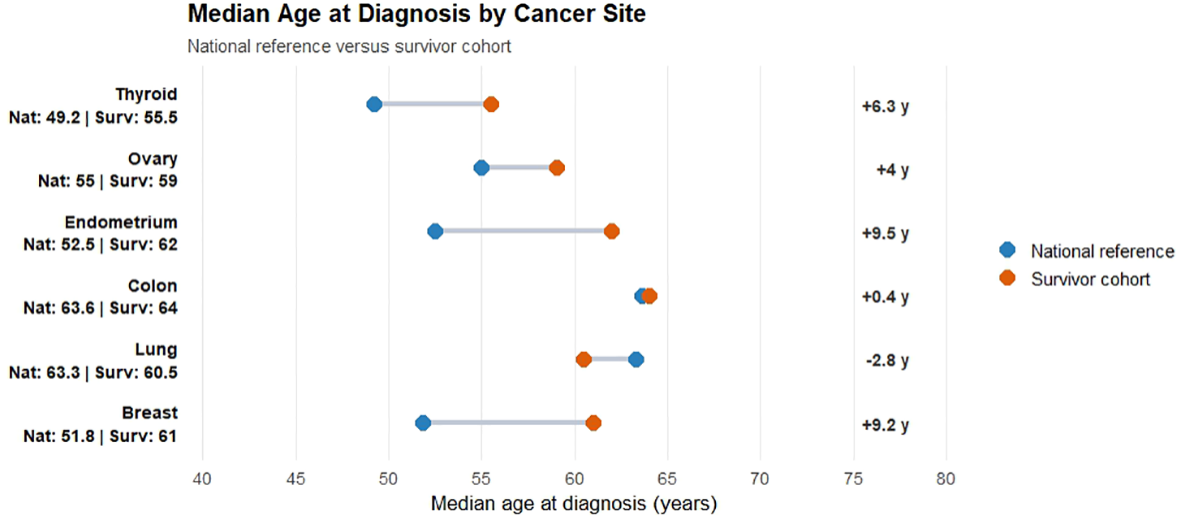
Median age at diagnosis of major second primary malignancies in the survivor cohort compared with national reference values. Blue circles indicate the national reference median age at diagnosis, whereas orange circles indicate the median age at diagnosis in the survivor cohort. The connecting line represents the difference between the two values. Labels on the left show the corresponding national and survivor median ages for each cancer site, and labels on the right indicate the absolute age difference.

**Figure 4:**
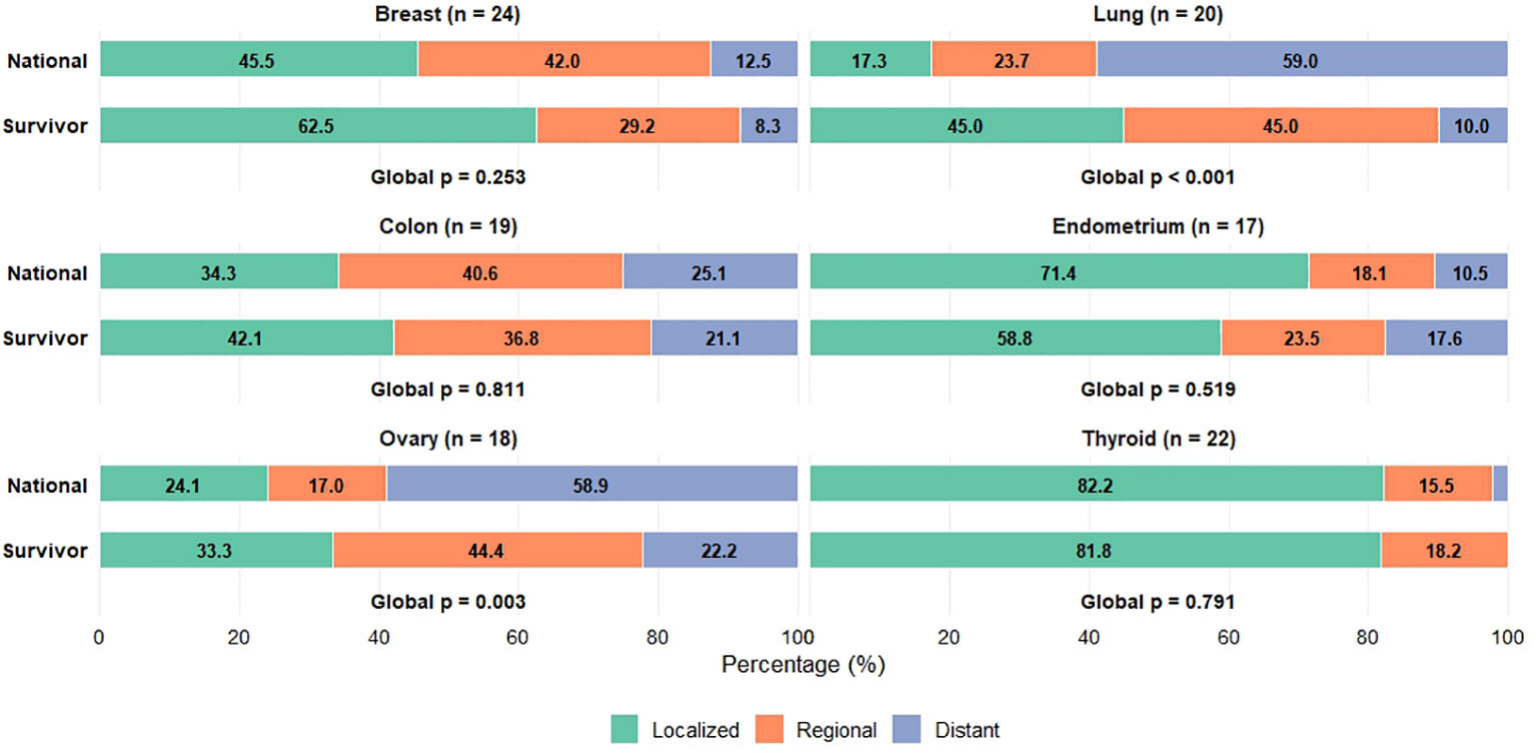
Three-category stage distribution of major second primary malignancies in the survivor cohort compared with national reference distributions. Stacked horizontal bars show the proportions of localized, regional, and distant disease at diagnosis for each cancer site. The upper bar in each panel represents the national reference distribution, and the lower bar represents the survivor cohort. Global p values were obtained using one-sample goodness-of-fit tests comparing the observed stage distribution in the survivor cohort with the corresponding national reference distribution.

**Figure 5:**
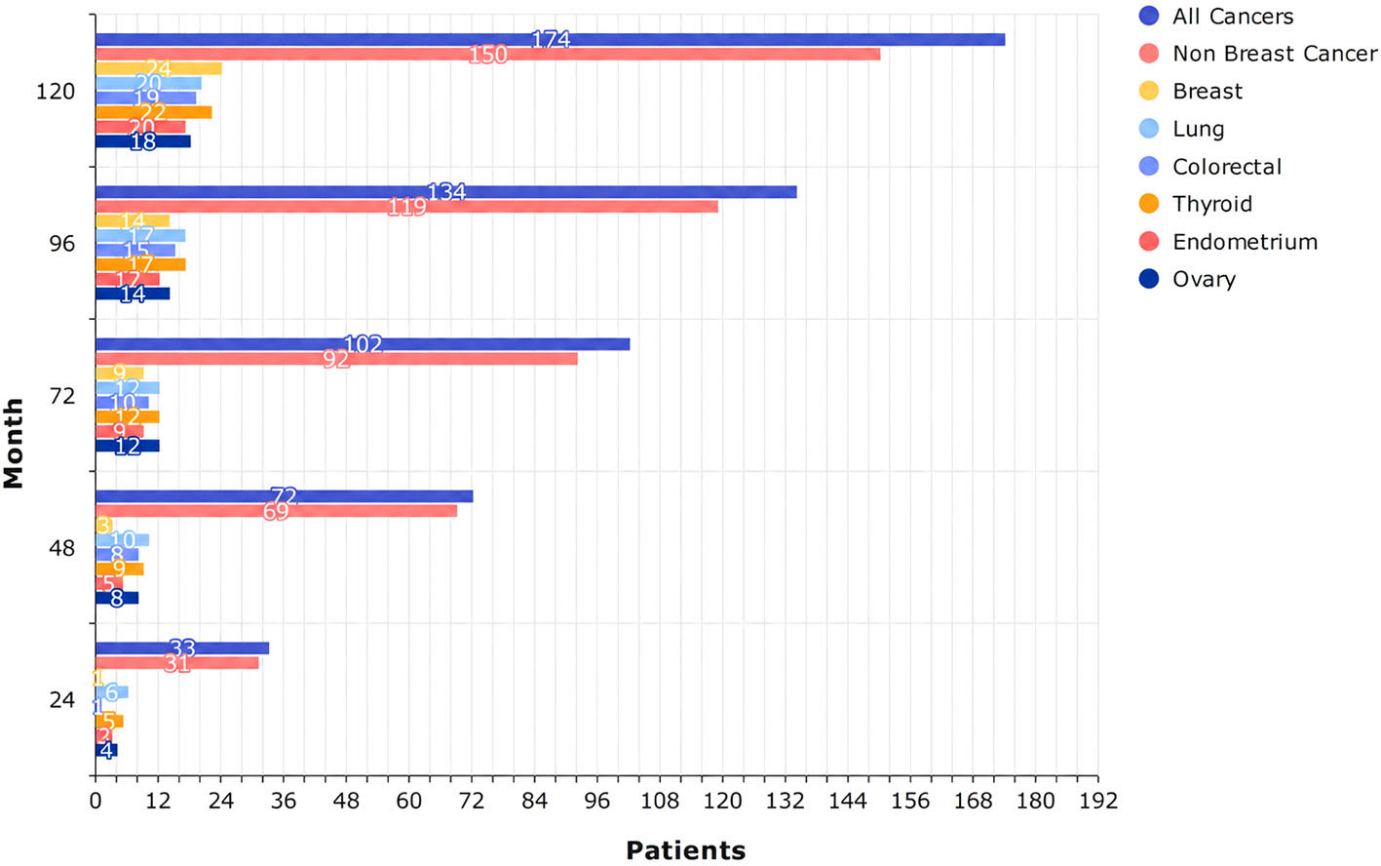
Cumulative counts of SPMs among breast cancer survivors across follow-up intervals (24–120 months): all SPMs, non-breast SPMs, and site-specific (lung, colorectal, thyroid, endometrium, ovary). Values on bars indicate patient numbers.

The median age at diagnosis in the survivor cohort was higher than the SEER population-based reference values for breast, endometrial, ovarian, and thyroid cancers, similar for colon cancer, and slightly lower for lung cancer (**Figure 3**). Because comparable age-stratified Turkish population-level stage distributions were not available, we used SEER population-based stage distributions as an external reference. To address the potential effect of surveillance bias, we performed an exploratory age-adjusted comparison of the three-category stage distribution (localized/regional/distant) for major SPMs. Significant differences were observed for lung cancer (global p<0.001) and ovarian cancer (global p=0.002), whereas no significant differences were found for breast, colon, endometrial, or thyroid cancers (**Figure 4**; **Supplementary Table 1**). Lung cancers in the survivor cohort were more frequently diagnosed at localized or regional stages and less frequently at distant stage than expected, while ovarian cancers showed a lower proportion of distant-stage disease and a higher proportion of regional-stage disease. The clinicopathological characteristics of SPMs occurring in 10 or more patients are presented in **Table 2** Comparisons between cancer subgroups revealed no statistically significant differences in age at diagnosis, T stage, nodal status, overall stage, histological subtype, HER2 status, ER status, chemotherapy regimen, radiotherapy administration, or time interval from primary breast cancer to the SPM2s (all p > 0.05). The median age at secondary cancer diagnosis ranged from 55.5 years in patients with thyroid cancer to 64.0 years in those with colorectal cancer (p = 0.124). Across subgroups, the majority presented with T2 stage disease and N0 or N1 nodal status. Stage II disease predominated in all groups, with IDC as the primary histological subtype, accounting for more than 80% of cases in each subgroup. HER2 positivity varied from 10.5% (colorectal cancer) to 37.5% (breast cancer), while ER positivity ranged from 55.6% (ovarian cancer) to 94.1% (endometrial cancer). Despite these variations, none of the differences reached statistical significance. **Figure 5**) (**Supplementary Table 3**).

**Table 2:** Clinicopathological characteristics of SPM’s with ≥10 cases and comparison across cancer subtypes.

Secondary tumour	Breast	Lung	Colorectal	Endometrium	Ovarian	Thyroid	Total	p
Total N (%)	24 (20.0)	20 (16.7)	19 (15.8)	17 (14.2)	18 (15.0)	22 (18.3)	120	
Age Median (IQR)	61.0 (44.5- 68.0)	60.5 (55.5-67.5)	64.0 (61.5-69.0)	62.0 (55.0-74.0)	59.0 (50.8-64.2)	55.5 (50.2-62.0)	61.0 (53.0-68.0)	0.068*
Stage								
Stage I	5 (20.8)	6 (30.0)	4 (21.1)	2 (11.8)	4 (22.2)	3 (13.6)	24 (20.0)	
Stage II	14 (58.3)	11 (55.0)	12 (63.2)	12 (70.6)	12 (66.7)	14 (63.6)	75 (62.5)	0.964**
Stage III	5 (20.8)	3 (15.0)	3 (15.8)	3 (17.6)	2 (11.1)	5 (22.7)	21 (17.5)	
Grade								
Grade 1	1 (4.2)	1 (5.0)	3 (15.8)	2 (11.8)	0 (0.0)	1 (4.5)	8 (6.7)	
Grade 2	13 (54.2)	12 (60.0)	14 (73.7)	6 (35.3)	13 (72.2)	16 (72.7)	74 (61.7)	0.146**
Grade 3	10 (41.7)	7 (35.0)	2 (10.5)	9 (52.9)	5 (27.8)	5 (22.7)	38 (31.7)	
Histological type								
IDC	24 (100.0)	18 (90.0)	12 (63.2)	17 (100.0)	16 (88.9)	18 (81.8)	105 (87.5)	
ILC	0 (0.0)	1 (5.0)	3 (15.8)	0 (0.0)	1 (5.6)	3 (13.6)	8 (6.7)	
Medullary	0 (0.0)	1 (5.0)	1 (5.3)	0 (0.0)	0 (0.0)	0 (0.0)	2 (1.7)	0.253**
Mucinous	0 (0.0)	0 (0.0)	2 (10.5)	0 (0.0)	1 (5.6)	1 (4.5)	4 (3.3)	
Other	0 (0.0)	0 (0.0)	1 (5.3)	0 (0.0)	0 (0.0)	0 (0.0)	1 (0.8)	
HER2								
HER2 IHC +	9 (37.5)	6 (30.0)	2 (10.5)	6 (35.3)	5 (27.8)	3 (13.6)	31 (25.8)	
HER2+ FISH-	0 (0.0)	2 (10.0)	0 (0.0)	1 (5.9)	2 (11.1)	1 (4.5)	6 (5.0)	0.403**
HER2+ FISH+	2 (8.3)	1 (5.0)	4 (21.1)	0 (0.0)	0 (0.0)	2 (9.1)	8 (6.7)	
Negative	13 (54.2)	11 (55.0)	13 (68.4)	10 (58.8)	11 (61.1)	16 (72.7)	74 (61.7)	
ER status								
Negative	3 (12.5)	3 (15.0)	4 (21.1)	1 (5.9)	4 (22.2)	3 (13.6)	18 (15.0)	
ER positive	20 (83.3)	16 (80.0)	14 (73.7)	16 (94.1)	10 (55.6)	18 (81.8)	94 (78.3)	0.269**
Unknown	1 (4.2)	1 (5.0)	1 (5.3)	0 (0.0)	4 (22.2)	1 (4.5)	8 (6.7)	
Treatment regimen								
Docetaxel Carboplatin	2 (8.3)	1 (5.0)	0 (0.0)	1 (5.9)	1 (5.6)	2 (9.1)	7 (5.8)	
Taxan	1 (4.2)	0 (0.0)	0 (0.0)	1 (5.9)	0 (0.0)	0 (0.0)	2 (1.7)	
FEC	4 (16.7)	3 (15.0)	3 (15.8)	1 (5.9)	2 (11.1)	4 (18.2)	17 (14.2)	0.885**
AC-Taxan	14 (48.4)	8 (40.0)	11 (57.9)	9 (52.8)	7 (38.9)	8 (57.1)	44 (51.7)	
AC	3 (12.5)	8 (40.0)	5 (26.3)	5 (29.4)	8 (44.4)	3 (13.6)	32 (26.7)	
Radiotherapy								
Yes	15 (62.5)	16 (80.0)	11 (57.9)	14 (82.4)	13 (72.2)	19 (86.4)	88 (73.3)	0.521**
No	8 (33.3)	4 (20.0)	7 (36.8)	3 (17.6)	5 (27.8)	2 (9.1)	29 (24.2)	
Time to SPM Median (IQR)	79.1 (60.9- 106.5)	69.4 (25.7- 97.4)	52.9 (24.4- 79.7)	48.7 (22.2- 76.1)	64.3 (30.9- 86.0)	60.9 (24.6- 82.9)	64.4 (25.9-88.2)	0.130*

SPM, Secondary primary malignancy; IDC, Invasive ductal carcinoma; ILC, Invasive lobular carcinoma; FEC, 5-fluorouracil, epirubicin, cyclophosphamide; AC, doxorubicin, cyclophosphamide; AC-Taxan, doxorubicin, cyclophosphamide followed by taxane; ER, estrogen receptor; HER2, human epidermal growth factor receptor 2; IQR, interquartile range. P-values were calculated using the Kruskal–Wallis test or one-way ANOVA for continuous variables, and the chi-square test or Fisher’s exact test for categorical variables, as appropriate.

## 4 Discussion

In this large, multicenter national cohort of chemotherapy-treated early-stage breast cancer survivors in Türkiye, we observed a significantly increased risk of developing SPMs compared with the general female population (SIR 1.66; 95% CI, 1.42–1.93). This excess risk remained elevated after excluding breast cancers (SIR 1.78; 95% CI, 1.51–2.09). These findings are consistent with, and extend, previous registry-based studies reporting a 28–62% higher risk of non-breast SPMs among breast cancer survivors. Notably, our study provides the first robust, large-scale estimates of SPM risk in Turkish breast cancer survivors, addressing an important gap in the literature from this region. Importantly, our results primarily reflect the risk of SPMs occurring within the first decade after breast cancer diagnosis, corresponding to the most intensively monitored period of survivorship in routine clinical practice (8, 9).

The development of second primary malignancies among breast cancer survivors is likely influenced by multiple factors, including tumour biology, baseline patient characteristics, treatment exposure, and treatment intensity. In the SEER-based analysis by Li et al., the overall risk of second primary cancers in the general breast cancer population was found to be broadly comparable to that of the background population (21). Likewise, the 25-year cohort analysis by Allen et al. reported SIR estimates that were generally consistent with population-level expectations (5). However, the Kaiser Permanente Breast Cancer Survivors Cohort showed that excess risks were more pronounced in selected subgroups, particularly patients with stage III disease and premenopausal breast cancer (8). Our cohort differs from unselected breast cancer survivor populations because all patients received chemotherapy and most also underwent radiotherapy, reflecting a more treatment-intensive and potentially higher-risk population. Accordingly, the elevated SIRs observed in the present study should be interpreted in the context of treatment selection and disease-risk enrichment, and may be more comparable to the higher-risk, chemotherapy-treated subgroups reported in the Kaiser Permanente cohort (8).The highest relative risks in our cohort were observed for thyroid cancer (SIR 7.09) and sarcomas (SIR 7.14). Although these findings may be biologically plausible in the context of prior anticancer treatment, including radiotherapy, the absence of detailed radiotherapy fields and dose information precludes a direct causal interpretation. Previous studies have documented radiation-associated angiosarcomas as rare but serious late complications of breast cancer treatment, and prior reviews have reported modestly elevated risks of thyroid cancer and sarcomas among breast cancer survivors, with thyroid cancer SIRs generally ranging from 1.29 to 1.79 and approximately twofold increases for sarcomas (9–11, 22–24). The higher estimates observed in our cohort may reflect the treatment-intensive nature of the study population, as most patients received multimodal therapy; however, alternative explanations should also be considered. In particular, the markedly increased thyroid cancer risk may have been influenced by intensified imaging-based surveillance, incidental detection of subclinical thyroid nodules, and regional background thyroid disease. The association between breast and thyroid cancers may also reflect a bidirectional relationship, as Li et al. reported an increased risk of second primary breast cancer after several first primary cancers, including thyroid cancer, suggesting shared endocrine, genetic, or surveillance-related determinants (25). Nevertheless, the magnitude of thyroid cancer excess in our cohort was substantially higher than the reciprocal risk reported in previous analyses, indicating that factors beyond shared biological susceptibility, including surveillance intensity, radiotherapy exposure, incidental detection, and regional thyroid disease background, may have contributed to the observed excess. Therefore, the elevated risks of thyroid cancer and sarcoma should be interpreted as multifactorial rather than as direct evidence of treatment-induced malignancy.We also observed a significantly increased risk of ovarian cancer (SIR 4.35), consistent with hereditary breast and ovarian cancer syndromes, particularly among BRCA1/2 mutation carriers (26). Recent studies indicate an increased risk of ovarian cancer among breast cancer survivors, with standardised incidence ratios (SIRs) of approximately 1.5–1.7; comparable estimates have been reported in other cohorts (8). Notably, among younger patients and those with estrogen receptor–negative disease, markedly higher risks have been observed, with SIRs ranging from 4.35 to 4.90 (27, 28). Although genetic testing data were not available for most patients in our study, the magnitude of this risk strongly supports the systematic referral of high-risk survivors for genetic counselling and testing (29). The modestly elevated risks of uterine corpus cancer and leukaemia in breast cancer survivors could be related to treatment exposures – tamoxifen therapy has been linked to secondary endometrial cancer, and specific chemotherapeutic agents (e.g., alkylators) can increase leukaemia risk (30, 31).Exposure to alkylating chemotherapy has been consistently linked to therapy-related myeloid neoplasms, including acute myeloid leukaemia and myelodysplastic syndromes, typically characterized by a latency period of 5–10 years and chromosomal abnormalities involving chromosomes 5 and 7 (31, 32). Likewise, the slight increase in lung cancer risk may reflect shared risk factors such as smoking or treatment-induced pulmonary effects.

In tumours other than those with increased risks, no significant differences were observed for gastric, brain, bladder, or colorectal cancers. The small numbers for specific sites—kidney (n = 3), bladder (n = 2), and brain (n = 2)—likely limited statistical power and may explain the absence of clinical significance, underscoring the need for larger or population-based studies. For cancer subtypes with small case numbers (e.g., pancreas, brain), the corresponding confidence intervals were wide, reflecting limited precision; therefore, these estimates should be interpreted with caution in a clinical context. Meta-analyses have similarly found no increase in the incidence of brain cancers and a slight increase in bladder cancer, a pattern that is also observed in Western cohorts (8, 10). In our treated cohort, the lack of an elevated SIR for contralateral breast cancer may reflect the effectiveness of contemporary systemic and locoregional therapies (33). The non-significant increase observed for colorectal cancer is consistent with reports showing that, particularly among women aged ≥50 years with a prior breast cancer diagnosis, risk elevations often do not reach statistical significance—potentially influenced by lifestyle and behavioural factors (34).In studies conducted in our country, major modifiable risk factors such as smoking, obesity, and alcohol consumption have been consistently highlighted, and their roles in carcinogenesis are well established (35). However, the endemic presence of goitre in the Black Sea region—which constitutes approximately 23.6% of our cohort—may have also contributed to the observed findings, particularly those related to thyroid cancer. Indeed, a large-scale study from this iodine-deficient endemic region demonstrated that papillary thyroid carcinoma is the predominant subtype, with the majority of cases diagnosed at an early stage, suggesting a more indolent tumour biology potentially associated with iodine supplementation (36). On the other hand, genetic factors may also play a contributory role, particularly in certain tumour subgroups. The relatively high rate of consanguineous marriages in Türkiye may lead to an increased prevalence of autosomal recessive disorders and genetic predispositions (37). Although the association between consanguinity and cancer risk remains inconsistent and controversial in the literature, it is plausible that genetic susceptibility may contribute to the development of rare tumours such as sarcomas, and consanguinity may indirectly influence this risk (38–40). Therefore, the interpretation of our findings should not rely solely on comparisons with global populations but should also account for region-specific environmental exposures and genetic characteristics.

The observed excess of SPMs may partly reflect intensified surveillance among breast cancer survivors. Because age-stratified Turkish population-level stage distributions were not available, we used SEER population-based stage distributions as an external reference and performed an exploratory age-adjusted stage comparison (41).As a complementary assessment, we also compared our findings with available Turkish stage distributions that were not stratified by age, and the overall pattern was broadly similar. This interpretation is supported by the finding that lung and ovarian cancers, which are typically diagnosed at advanced stages in the general population, were identified at relatively earlier stages in our cohort (14, 16). Indeed, lung and ovarian cancers showed significantly different stage distributions compared with the SEER reference population, with fewer distant-stage diagnoses and relatively more localized or regional-stage presentations. However, this explanation does not appear to apply uniformly across all malignancies. For example, the stage distribution of thyroid cancer was broadly comparable with the SEER population-based reference distribution, while the age at diagnosis was higher than expected, arguing against surveillance alone as the sole explanation for the excess risk. Similarly, no significant age-adjusted stage-distribution differences were observed for contralateral breast, colorectal, or endometrial cancers. In addition, the SIR estimates for several tumour types were broadly consistent with those reported in previous studies (20). Taken together, these findings suggest that surveillance bias may have contributed to some of the observed excess, particularly for malignancies that could plausibly be detected through real-world follow-up imaging, such as chest imaging and abdominal ultrasonography, but is unlikely to fully account for the overall pattern. These comparisons should nevertheless be interpreted cautiously, as they were based on external reference data rather than individually matched controls. Our findings may inform personalised survivorship care planning by supporting a risk-adapted rather than uniform approach to follow-up. Given the elevated relative risks observed for thyroid cancer, ovarian cancer, and sarcomas, clinicians should remain attentive to site-specific symptoms and incidental abnormalities detected during routine survivorship care. Importantly, the increased thyroid cancer SIR should not be interpreted as evidence supporting universal thyroid screening for all breast cancer survivors. Rather, thyroid findings identified during clinical evaluation or imaging may warrant careful assessment, particularly among patients with prior regional radiotherapy or from regions with a high background prevalence of thyroid disease. The increased ovarian cancer risk further supports the integration of genetic risk assessment and referral for counselling when clinically indicated. Together with counselling on modifiable lifestyle factors, these approaches may contribute to more individualized and evidence-informed survivorship care (42, 43).

The strengths of our study include its multicentre design, large national sample, and pathologically confirmed diagnoses of second primary malignancies (SPMs). Additionally, the use of contemporary national cancer registry incidence rates enabled age-, sex-, and calendar year-standardised calculations, thereby enhancing the accuracy of standardised incidence ratio (SIR) estimates. However, several limitations should be acknowledged. Incomplete clinicopathologic data for patients without SPMs limited subgroup and adjusted analyses. Another important limitation is the absence of an individual-level breast cancer-free comparator cohort, precluding age-adjusted Cox regression analyses when comparing age at diagnosis between breast cancer survivors and the general population. Although stage distributions were primarily compared using national reference data, age-stratified Turkish population-level stage distributions were unavailable. Therefore, the exploratory age-adjusted stage analyses presented in **Supplementary Table 2** relied on SEER population-based reference distributions and should be interpreted cautiously. Variations in follow-up intensity across centres may have introduced surveillance bias, particularly for malignancies detectable through routine imaging; however, stage-distribution analyses suggested that this effect was not uniform across all tumour types. The absence of vital status, cause-of-death information, detailed treatment exposure data, individual-level risk factors, and systematic germline testing limited survival analyses and adjustment for potential confounders. Finally, incomplete capture of SPMs diagnosed outside participating centres may have resulted in slight underestimation of true SIRs.

## 5 Conclusions

Despite these limitations, our study provides the first large-scale, real-world data on SPM risk among breast cancer survivors in Türkiye. However, these findings should be interpreted in the context of treatment selection, surveillance intensity, hereditary predisposition, regional background risks, and the absence of detailed treatment-exposure data. Rather than implying a direct causal effect of chemotherapy, our results highlight the need for more refined survivorship research incorporating systematic germline testing, treatment-level exposure data, and standardized follow-up. Such studies are required to develop evidence-based, risk-adapted survivorship strategies for breast cancer survivors in Türkiye.

## Data Availability

The raw data supporting the conclusions of this article will be made available by the authors, without undue reservation.
