# Supplementary material for: Ten-year risk of second primary malignancies among chemotherapy-treated early-stage breast cancer survivors: a multicentre cohort study from the Turkish Oncology Group
Source: Front Oncol. 2026 Jul 7;16:1756315. doi: 10.3389/fonc.2026.1756315 (PMC13384881; doi:10.3389/fonc.2026.1756315)
Supplement: Supplementary file 1 [file Table1.docx]

**Suplemantary Table 1:** Three-category stage distribution of major second primary malignancies in the survivor cohort compared with national reference distributions

| Cancer site | Survivor cohort, n | National stage distribution, % (localized/regional/distant) | Survivor stage distribution, n (%) (localized/regional/distant) | p value |
| --- | --- | --- | --- | --- |
| Breast | 24 | 45.5 / 42.0 / 12.5 | 13 (54.2) / 9 (37.5) / 2 (8.3) | 0.655 |
| Lung | 20 | 17.3 / 23.7 / 59.0 | 9 (45.0) / 9 (45.0) / 2 (10.0) | <0.001 |
| Colon | 19 | 34.3 / 40.6 / 25.1 | 8 (42.1) / 7 (36.8) / 4 (21.1) | 0.811 |
| Endometrium | 17 | 71.4 / 18.1 / 10.5 | 10 (58.8) / 4 (23.5) / 3 (17.6) | 0.519 |
| Ovary | 18 | 24.1 / 17.0 / 58.9 | 6 (33.3) / 8 (44.4) / 4 (22.2) | 0.003 |
| Thyroid | 22 | 82.2 / 15.5 / 2.3 | 16 (72.7) / 4 (18.2) / 2 (9.1) | 0.093 |

**Footnote:** Global p values were obtained using one-sample goodness-of-fit tests comparing the observed three-category stage distribution in the survivor cohort with the national reference distribution for each cancer site.

**Supplementary Table 2.** Age-standardized observed-to-expected comparison of stage distribution for major second primary malignancies using external population-based reference stage distributions.

| **Cancer** | **Age group** | **Total n** | **Obs. localized** | **Exp. localized** | **O/E** | **Obs. regional** | **Exp. regional** | **O/E** | **Obs. distant** | **Exp. distant** | **O/E** |
| --- | --- | --- | --- | --- | --- | --- | --- | --- | --- | --- | --- |
| Breast | <50 | 9 | 4 | 5.1 | 0.78 | 4 | 3.1 | 1.31 | 1 | 0.6 | 1.59 |
| Breast | 50–64 | 5 | 2 | 3.2 | 0.62 | 2 | 1.4 | 1.43 | 1 | 0.3 | 3.33 |
| Breast | ≥65 | 10 | 7 | 6.7 | 1.04 | 3 | 2.5 | 1.20 | 0 | 0.5 | 0.00 |
| Lung | <50 | 4 | 2 | 0.7 | 2.78 | 2 | 0.9 | 2.27 | 0 | 2.2 | 0.00 |
| Lung | 50–64 | 8 | 4 | 1.8 | 2.27 | 3 | 1.8 | 1.70 | 1 | 4.2 | 0.24 |
| Lung | ≥65 | 8 | 3 | 2.0 | 1.50 | 4 | 1.7 | 2.38 | 1 | 3.9 | 0.26 |
| Colorectal | <50 | 0 | 0 | 0.0 | — | 0 | 0.0 | — | 0 | 0.0 | — |
| Colorectal | 50–64 | 9 | 4 | 2.9 | 1.39 | 3 | 3.4 | 0.88 | 2 | 2.2 | 0.93 |
| Colorectal | ≥65 | 10 | 4 | 3.6 | 1.11 | 4 | 3.6 | 1.11 | 2 | 2.1 | 0.95 |
| Endometrium/Uterus | <50 | 5 | 3 | 3.6 | 0.83 | 1 | 0.8 | 1.33 | 1 | 0.4 | 2.50 |
| Endometrium/Uterus | 50–64 | 7 | 4 | 4.9 | 0.82 | 2 | 1.2 | 1.68 | 1 | 0.6 | 1.59 |
| Endometrium/Uterus | ≥65 | 5 | 3 | 3.1 | 0.97 | 1 | 1.0 | 1.00 | 1 | 0.7 | 1.54 |
| Ovary | <50 | 5 | 2 | 1.4 | 1.43 | 2 | 1.0 | 2.11 | 1 | 2.2 | 0.44 |
| Ovary | 50–64 | 9 | 3 | 1.9 | 1.59 | 4 | 1.6 | 2.47 | 2 | 5.0 | 0.40 |
| Ovary | ≥65 | 4 | 1 | 0.7 | 1.47 | 2 | 0.7 | 2.94 | 1 | 2.4 | 0.42 |
| Thyroid | <50 | 11 | 8 | 7.2 | 1.12 | 2 | 3.4 | 0.59 | 1 | 0.2 | 4.55 |
| Thyroid | 50–64 | 6 | 4 | 3.7 | 1.08 | 1 | 1.9 | 0.54 | 1 | 0.2 | 4.17 |
| Thyroid | ≥65 | 5 | 4 | 2.8 | 1.45 | 1 | 1.5 | 0.67 | 0 | 0.4 | 0.00 |

Footnote :Expected counts were calculated by multiplying the number of observed SPMs in each cancer-specific age stratum by the corresponding age-specific reference stage proportions. O/E, observed-to-expected ratio. This analysis was exploratory because individual-level comparator data and reference denominators were unavailable

**Supplementary Table 3.** Clinical and pathological characteristics according to second primary malignancy subtype

| **Variable** |  | **Brain**  **(n=2)** | **Thyroid**  **(n=22)** | **Bladder**  **(n=2)** | **Sarcoma**  **(n=14)** | **Stomach**  **(n=7)** | **Head & Neck (n=6)** | **Leukemia**  **(n=10)** | **Kidney**  **(n=3)** | **Lymphoma**  **(n=2)** | **Pancreas**  **(n=4)** | **Skin**  **(n=5)** | **Ovary**  **(n=18)** | **Endometrium (n=17)** | **Colorectal**  **(n=18)** | **Lung**  **(n=20)** | **Breast**  **(n=24)** | **Total**  **(n=174)** | ***p*-value** |
| --- | --- | --- | --- | --- | --- | --- | --- | --- | --- | --- | --- | --- | --- | --- | --- | --- | --- | --- | --- |
| **Age** | **Median** | 57.5 | 55.5 | 56.5 | 59.5 | 64.0 | 59.0 | 62.5 | 73.0 | 67.5 | 63.0 | 64.0 | 59.0 | 62.0 | 65.5 | 60.5 | 61.0 | 62.0 | 0.437 |
| **Stage at diagnosis** | **I** | 0  (0.0) | 3 (13.6) | 0 (0.0) | 1  (7.1) | 2 (28.6) | 2 (33.3) | 2 (20.0) | 0  (0.0) | 0  (0.0) | 1 (25.0) | 1 (20.0) | 4 (22.2) | 2 (12.5) | 4 (23.5) | 6 (30.0) | 5 (20.8) | 33 (19.3) | 0.538 |
|  | **II** | 2 (100) | 14 (63.6) | 0 (0.0) | 13 (92.9) | 3 (42.9) | 3 (50.0) | 8 (80.0) | 1 (50.0) | 1 (50.0) | 3 (75.0) | 3 (60.0) | 12 (66.7) | 11 (68.8) | 10 (58.8) | 11 (55.0) | 14 (58.3) | 109 (63.7) |  |
|  | **III** | 0  (0.0) | 5 (22.7) | 2 (100) | 0  (0.0) | 2 (28.6) | 1 (16.7) | 0  (0.0) | 1 (50.0) | 1 (50.0) | 0  (0.0) | 1 (20.0) | 2 (11.1) | 3 (18.8) | 3 (17.6) | 3 (15.0) | 5 (20.8) | 29 (17.0) |  |
| **T Stage** | **T1** | 0  (0.0) | 10 (45.5) | 0 (0.0) | 2 (14.3) | 2 (28.6) | 2 (33.3) | 3 (30.0) | 0  (0.0) | 0  (0.0) | 1 (25.0) | 3 (60.0) | 8 (44.4) | 7 (43.8) | 8 (44.4) | 7 (35.0) | 6 (25.0) | 59 (34.3) | 0.879 |
|  | **T2** | 2 (100) | 11 (50.0) | 2 (100) | 11 (78.6) | 5 (71.4) | 3 (50.0) | 7 (70.0) | 2 (100) | 2 (100) | 3 (75.0) | 2 (40.0) | 10 (55.6) | 9 (56.2) | 9 (50.0) | 13 (65.0) | 17 (70.8) | 108 (62.8) |  |
|  | **T3** | 0  (0.0) | 1  (4.5) | 0 (0.0) | 1  (7.1) | 0  (0.0) | 1 (16.7) | 0  (0.0) | 0  (0.0) | 0  (0.0) | 0  (0.0) | 0  (0.0) | 0  (0.0) | 0  (0.0) | 1 (5.6) | 0  (0.0) | 1 (4.2) | 5 (2.9) |  |
| **N Stage** | **N0** | 2 (100) | 10 (45.5) | 0 (0.0) | 7 (50.0) | 3 (42.9) | 4 (66.7) | 3 (30.0) | 1 (33.3) | 1 (50.0) | 3 (75.0) | 1 (20.0) | 7 (38.9) | 7 (41.2) | 9 (50.0) | 11 (55.0) | 13 (54.2) | 82 (47.1) | 0.400 |
|  | **N1** | 0  (0.0) | 7 (31.8) | 0 (0.0) | 7 (50.0) | 2 (28.6) | 2 (33.3) | 7 (70.0) | 1 (33.3) | 0  (0.0) | 1 (25.0) | 3 (60.0) | 9 (50.0) | 7 (41.2) | 6 (33.3) | 6 (30.0) | 6 (25.0) | 64 (36.8) |  |
|  | **N2** | 0  (0.0) | 3 (13.6) | 1 (50.0) | 0  (0.0) | 0  (0.0) | 0  (0.0) | 0  (0.0) | 1 (33.3) | 1 (50.0) | 0  (0.0) | 0  (0.0) | 1  (5.6) | 1  (5.9) | 2 (11.1) | 1  (5.0) | 4 (16.7) | 15 (8.6) |  |
| **Tumor grade** | **1** | 0  (0.0) | 1  (4.5) | 0 (0.0) | 0  (0.0) | 0  (0.0) | 0  (0.0) | 0  (0.0) | 0  (0.0) | 0  (0.0) | 0  (0.0) | 0  (0.0) | 0  (0.0) | 2 (11.8) | 3 (16.7) | 1  (5.0) | 1 (4.2) | 8 (4.6) | 0.078 |
|  | **2** | 1 (50.0) | 16 (72.7) | 0 (0.0) | 4 (28.6) | 3 (42.9) | 5 (83.3) | 6 (60.0) | 3 (100) | 0  (0.0) | 2 (50.0) | 1 (20.0) | 13 (72.2) | 6 (35.3) | 13 (72.2) | 12 (60.0) | 13 (54.2) | 98 (56.3) |  |
|  | **3** | 1 (50.0) | 5 (22.7) | 2 (100) | 10 (71.4) | 4 (57.1) | 1 (16.7) | 4 (40.0) | 0  (0.0) | 2 (100) | 2 (50.0) | 4 (80.0) | 5 (27.8) | 9 (52.9) | 2 (11.1) | 7 (35.0) | 10 (41.7) | 68 (39.1) |  |
| **Histology** | **IDC** | 2 (100) | 18 (81.8) | 2 (100) | 13 (92.9) | 6 (85.7) | 5 (83.3) | 8 (80.0) | 3 (100) | 1 (50.0) | 4 (100) | 5 (100) | 16 (88.9) | 17 (100) | 11 (61.1) | 18 (90.0) | 24 (100) | 153 (87.9) | 0.278 |
|  | **ILC** | 0  (0.0) | 3 (13.6) | 0 (0.0) | 1  (7.1) | 1 (14.3) | 1 (16.7) | 0  (0.0) | 0  (0.0) | 0  (0.0) | 0  (0.0) | 0  (0.0) | 1  (5.6) | 0  (0.0) | 3 (16.7) | 1  (5.0) | 0 (0.0) | 11 (6.3) |  |
| **ER status** | **Positive** | 2 (100) | 18 (81.8) | 2 (100) | 11 (78.6) | 5 (71.4) | 6 (100) | 7 (70.0) | 3 (100) | 1 (50.0) | 3 (75.0) | 5 (100) | 10 (55.6) | 16 (94.1) | 13 (72.2) | 16 (80.0) | 20 (83.3) | 138 (79.3) | 0.699 |
|  | **Negative** | 0  (0.0) | 3 (13.6) | 0 (0.0) | 2 (14.3) | 2 (28.6) | 0  (0.0) | 3 (30.0) | 0  (0.0) | 1 (50.0) | 1 (25.0) | 0  (0.0) | 4 (22.2) | 1  (5.9) | 4 (22.2) | 3 (15.0) | 3 (12.5) | 27 (15.5) |  |
| **HER 2 status** | **Negative** | 2 (100) | 16 (72.7) | 0 (0.0) | 11 (78.6) | 5 (71.4) | 5 (83.3) | 8 (80.0) | 2 (66.7) | 2 (100) | 3 (75.0) | 3 (60.0) | 11 (61.1) | 10 (58.8) | 12 (66.7) | 11 (55.0) | 13 (54.2) | 114 (65.5) | 0.857 |
|  | **IHC 1+** | 0  (0.0) | 2  (9.1) | 0 (0.0) | 0  (0.0) | 0  (0.0) | 0  (0.0) | 1 (10.0) | 1 (33.3) | 0  (0.0) | 0  (0.0) | 1 (20.0) | 0  (0.0) | 0  (0.0) | 3 (16.7) | 1  (5.0) | 2 (8.3) | 11 (6.3) |  |
|  | **IHC 2+ (FISH−)** | 0  (0.0) | 0  (0.0) | 0 (0.0) | 0  (0.0) | 0  (0.0) | 0  (0.0) | 0  (0.0) | 0  (0.0) | 0  (0.0) | 0  (0.0) | 0  (0.0) | 0  (0.0) | 0  (0.0) | 1 (5.6) | 0  (0.0) | 0 (0.0) | 1 (0.6) |  |
|  | **IHC 2+**  **FISH (+)** | 0  (0.0) | 1  (4.5) | 0 (0.0) | 1  (7.1) | 1 (14.3) | 0  (0.0) | 0  (0.0) | 0  (0.0) | 0  (0.0) | 0  (0.0) | 0  (0.0) | 2 (11.1) | 1  (5.9) | 0 (0.0) | 2 (10.0) | 0 (0.0) | 8 (4.6) |  |
|  | **IHC 3 +** | 0  (0.0) | 3 (13.6) | 2 (100) | 2 (14.3) | 1 (14.3) | 1 (16.7) | 1 (10.0) | 0  (0.0) | 0  (0.0) | 1 (25.0) | 1 (20.0) | 5 (27.8) | 6 (35.3) | 2 (11.1) | 6 (30.0) | 9 (37.5) | 40 (23.0) |  |
| **Chemotherapy** | **AC** | 1 (50.0) | 3 (13.6) | 0 (0.0) | 3 (21.4) | 0  (0.0) | 3 (50.0) | 1 (10.0) | 2 (66.7) | 0  (0.0) | 0  (0.0) | 3 (60.0) | 8 (44.4) | 5 (29.4) | 5 (27.8) | 8 (40.0) | 3 (12.5) | 45 (25.9) | 0.808 |
|  | **AC-Taxan** | 1 (50.0) | 8 (36.4) | 1 (50.0) | 7 (50.0) | 4 (57.1) | 0  (0.0) | 6 (60.0) | 1 (33.3) | 0  (0.0) | 1 (25.0) | 1 (20.0) | 6 (33.3) | 6 (35.3) | 7 (38.9) | 6 (30.0) | 10 (41.7) | 65 (37.4) |  |
|  | **FEC** | 0  (0.0) | 4 (18.2) | 0 (0.0) | 1  (7.1) | 1 (14.3) | 0  (0.0) | 1 (10.0) | 0  (0.0) | 1 (50.0) | 1 (25.0) | 0  (0.0) | 2 (11.1) | 1  (5.9) | 3 (16.7) | 3 (15.0) | 4 (16.7) | 22 (12.6) |  |
|  | **Taxan** | 0  (0.0) | 0  (0.0) | 0 (0.0) | 0  (0.0) | 0  (0.0) | 0  (0.0) | 0  (0.0) | 0  (0.0) | 0  (0.0) | 0  (0.0) | 0  (0.0) | 0  (0.0) | 1  (5.9) | 0 (0.0) | 0  (0.0) | 1 (4.2) | 2 (1.1) |  |
|  | **Docetaxel Carbo** | 0  (0.0) | 2  (9.1) | 1 (50.0) | 1  (7.1) | 0  (0.0) | 2 (33.3) | 2 (20.0) | 0  (0.0) | 1 (50.0) | 1 (25.0) | 0  (0.0) | 1  (5.6) | 1  (5.9) | 0 (0.0) | 1  (5.0) | 2 (8.3) | 15 (8.6) |  |
| **Radiotherapy** | **No** | 1 (50.0) | 2  (9.1) | 0 (0.0) | 2 (14.3) | 0  (0.0) | 2 (33.3) | 4 (40.0) | 0  (0.0) | 0  (0.0) | 1 (25.0) | 2 (40.0) | 5 (27.8) | 3 (17.6) | 7 (38.9) | 4 (20.0) | 8 (33.3) | 41 (23.6) | 0.139 |
|  | **Yes** | 0  (0.0) | 19 (86.4) | 2 (100) | 12 (85.7) | 6 (85.7) | 4 (66.7) | 6 (60.0) | 2 (66.7) | 2 (100) | 3 (75.0) | 3 (60.0) | 13 (72.2) | 13 (76.5) | 10 (55.6) | 16 (80.0) | 15 (62.5) | 126 (72.4) |  |

Abbreviations: IDC, invasive ductal carcinoma; ILC, invasive lobular carcinoma; ER, estrogen receptor; HER2, human epidermal growth factor receptor 2.
Values are presented as median (interquartile range [IQR]) or n (%).
Continuous variables were compared using one-way analysis of variance (ANOVA) following assessment of distributional assumptions; categorical variables were analyzed using the chi-square test or Fisher’s exact test, as appropriate.
All p-values are two-sided and reflect comparisons across second primary malignancy subtypes; where relevant, reported p-values correspond to pairwise comparisons between clinically comparable subgroups.
